# Supplementary material for: Geographically persistent clusters of La Crosse virus disease in the Appalachian region of the United States from 2003 to 2021
Source: PLoS Negl Trop Dis. 2023 Jan 19;17(1):e0011065. doi: 10.1371/journal.pntd.0011065 (PMC9888679; doi:10.1371/journal.pntd.0011065)
Supplement: Attachment — Submitted filename: PNTD-D-22-01013.pdf [file pntd.0011065.s001.pdf]

PLOS Neglected Tropical Diseases

Persistent high-risk geographic clusters of neuroinvasive La Crosse virus disease in the socioeconomically disadvantaged Appalachian region of the United States

--Manuscript Draft--

**Manuscript Number:**	PNTD-D-22-01013
**Full Title:**	Persistent high-risk geographic clusters of neuroinvasive La Crosse virus disease in the socioeconomically disadvantaged Appalachian region of the United States
**Short Title:**	Persistent risk clustering of neuroinvasive La Crosse virus disease
**Article Type:**	Research Article
**Keywords:**	Keywords: La Crosse encephalitis, La Crosse virus, Arboviral encephalitis, Risk Clustering, Persistent Risk, Risk predictors
**Abstract:**	La Crosse virus (LACV) is a mosquito-borne pathogen that causes more pediatric neuroinvasive disease than any other arbovirus in the United States. In the 1990s, the distribution of LACV disease expanded rapidly from the upper Midwest into southern Appalachia, but there is a scarcity of research that investigates continued changes in LACV disease distribution in recent decades. The objectives of this study were to: (1) investigate changes in the distribution of geographic clusters of neuroinvasive LACV disease between 2003 and 2021 and (2) investigate socioeconomic and demographic predictors of county-level disease risk in states with persistent high-risk clusters. We used flexible spatial scan statistics to identify high-risk clusters from 2003 to 2021 and a generalized mixed model to identify socioeconomic and demographic predictors of disease risk. We identified high-risk clusters in the Appalachian region, with persistent clusters in east Tennessee / western North Carolina, West Virginia, and Ohio. In those states, high disease risks occurred in counties where high percentages of the population were white or had a low median household income. The findings present clear evidence that neuroinvasive LACV disease risk is persistent in the socioeconomically disadvantaged Appalachian region.
**Additional Information:**	
**Question**	**Response**
**Financial Disclosure**<br><br>Enter a financial disclosure statement that describes the sources of funding for the work included in this submission. Review the submission guidelines for detailed requirements. View published research articles from *PLOS NTDs* for specific examples.<br><br>This statement is required for submission and **will appear in the published article** if the submission is accepted. Please make sure it is accurate.	The author(s) received no specific funding for this work.

Powered by Editorial Manager® and ProduXion Manager® from Aries Systems Corporation

**Unfunded studies**<br><br>Enter: *The author(s) received no specific funding for this work.*<br><br>**Funded studies**<br><br>Enter a statement with the following details:<br>• Initials of the authors who received each award<br>• Grant numbers awarded to each author<br>• The full name of each funder<br>• URL of each funder website<br>• Did the sponsors or funders play any role in the study design, data collection and analysis, decision to publish, or preparation of the manuscript?<br>• **NO** - Include this sentence at the end of your statement: *The funders had no role in study design, data collection and analysis, decision to publish, or preparation of the manuscript.*<br>• **YES** - Specify the role(s) played.<br><br>* typeset	
**Competing Interests**<br><br>Use the instructions below to enter a competing interest statement for this submission. On behalf of all authors, disclose any competing interests that could be perceived to bias this work—acknowledging all financial support and any other relevant financial or non-financial competing interests.<br><br>This statement **will appear in the published article** if the submission is accepted. Please make sure it is accurate. View published research articles from *PLOS NTDs* for specific examples.	The authors have declared that no competing interests exist.

**NO authors have competing interests**<br><br>Enter: *The authors have declared that no competing interests exist.*<br><br>**Authors with competing interests**<br><br>Enter competing interest details beginning with this statement:<br><br>*I have read the journal's policy and the authors of this manuscript have the following competing interests: [insert competing interests here]*<br><br>* typeset<br><br>This statement is required for submission and **will appear in the published article** if the submission is accepted. Please make sure it is accurate and that any funding sources listed in your Funding Information later in the submission form are also declared in your Financial Disclosure statement.	
**Data Availability**<br><br>Authors are required to make all data underlying the findings described fully available, without restriction, and from the time of publication. PLOS allows rare exceptions to address legal and ethical concerns. See the PLOS Data Policy and FAQ for detailed information.	Yes - all data are fully available without restriction

A Data Availability Statement describing where the data can be found is required at submission. Your answers to this question constitute the Data Availability Statement and **will be published in the article**, if accepted.<br><br>**Important:** Stating 'data available on request from the author' is not sufficient. If your data are only available upon request, select 'No' for the first question and explain your exceptional situation in the text box.<br><br>Do the authors confirm that all data underlying the findings described in their manuscript are fully available without restriction?	
**Describe where the data may be found in full sentences. If you are copying our sample text, replace any instances of XXX with the appropriate details.**<br><br>• If the data are **held or will be held in a public repository**, include URLs, accession numbers or DOIs. If this information will only be available after acceptance, indicate this by ticking the box below. For example: *All XXX files are available from the XXX database (accession number(s) XXX, XXX.*).<br>• If the data are all contained **within the manuscript and/or Supporting Information files**, enter the following: *All relevant data are within the manuscript and its Supporting Information files.*<br>• If neither of these applies but you are able to provide **details of access elsewhere**, with or without limitations, please do so. For example:<br><br>*Data cannot be shared publicly because*	All relevant data are within the Supporting Information files. We are willing to upload the data to a public repository upon acceptance of the manuscript.

of [XXX]. Data are available from the XXX Institutional Data Access / Ethics Committee (contact via XXX) for researchers who meet the criteria for access to confidential data.<br><br>*The data underlying the results presented in the study are available from (include the name of the third party and contact information or URL).*<br>• This text is appropriate if the data are owned by a third party and authors do not have permission to share the data.<br><br>* typeset	
Additional data availability information:	Tick here if the URLs/accession numbers/DOIs will be available only after acceptance of the manuscript for publication so that we can ensure their inclusion before publication.

Powered by Editorial Manager® and ProduXion Manager® from Aries Systems Corporation

Manuscript (.docx version)

Click here to access/download;Manuscript;PersistentLACVClusters_Submissi

[c] C. A. Day

Entomology & Plant Pathology Department

University of Tennessee - Knoxville

Knoxville, TN 37996

E-mail: cday11@vols.utk.edu

Persistent high-risk geographic clusters of neuroinvasive La Crosse virus disease in the socioeconomically disadvantaged Appalachian region of the United States

C.A. Day[1c], A. Odoi[2], and R.T. Trout Fryxell[1]

[1] Department of Entomology and Plant Pathology, University of Tennessee, Knoxville TN 37996

[2] Department of Biomedical and Diagnostic Sciences, University of Tennessee, Knoxville TN 37996

Abstract

La Crosse virus (LACV) is a mosquito-borne pathogen that causes more pediatric neuroinvasive disease than any other arbovirus in the United States. In the 1990s, the distribution of LACV disease expanded rapidly from the upper Midwest into southern Appalachia, but there is a scarcity of research that investigates continued changes in LACV disease distribution in recent decades. The objectives of this study were to: (1) investigate changes in the distribution of geographic clusters of neuroinvasive LACV disease between 2003 and 2021 and (2) investigate socioeconomic and demographic predictors of county-level disease risk in states with persistent high-risk clusters. We used flexible spatial scan statistics to identify high-risk clusters from 2003 to 2021 and a generalized mixed model to identify socioeconomic and demographic predictors of disease risk. We identified high-risk clusters in the Appalachian region, with persistent clusters in east Tennessee / western North Carolina, West Virginia, and Ohio. In those states, high disease risks occurred in counties where high percentages of the population were white or had a low median household income. The findings present clear evidence that neuroinvasive LACV disease risk is persistent in the socioeconomically disadvantaged Appalachian region.

Keywords: La Crosse encephalitis, La Crosse virus, Arboviral encephalitis, Risk Clustering, Persistent Risk,

Author Summary

La Crosse virus (LACV) is a mosquito-borne pathogen that causes neuroinvasive disease (e.g., encephalitis and meningitis) in humans. Most neuroinvasive LACV disease occurs in children, and LACV is responsible for more pediatric neuroinvasive disease than any other virus transmitted by arthropods in the United States. In the 1990s, the geographic focus of LACV disease shifted into the southeastern US, but there is a lack of information on how its distribution has changed in recent decades. Our study had two objectives: (1) investigate changes in the geographic distribution of high-risk clusters of neuroinvasive LACV disease from 2003 to 2021 and (2) identify socioeconomic and demographic factors that are associated with high disease risk. We found that high-risk clusters of neuroinvasive LACV disease risk had a consistent distribution from 2003 to 2021, with the Appalachian region containing high-risk clusters during the entire period. We also found that in states with persistent high-risk clusters, county-level disease risk was highest in counties with high proportions of white populations and when median household income was lower. These results indicate that neuroinvasive LACV disease risk is geographically persistent, particularly in disadvantaged Appalachian counties. The results will be useful for targeting efforts to reduce LACV disease.

Introduction

La Crosse virus (LACV) is a California serogroup virus transmitted by *Aedes* (Diptera: Culicidae) mosquitoes that use natural and artificial containers for immature development [1]. Initially isolated in 1964 from a deceased child in La Crosse, Wisconsin, LACV has since caused annual morbidity in the US and is responsible for more neuroinvasive pediatric disease than any other arbovirus in the country [2–6]. Neuroinvasive cases of LACV disease typically present as pediatric encephalitis, meningitis, or meningoencephalitis and often cause long-term neurologic sequelae such as reduced mental function and chronic seizure disorders [7–9] . Historically, LACV disease was mostly restricted to rural areas in the upper Midwest (i.e., Illinois, Iowa, Indiana, Minnesota, and Wisconsin) and Ohio [10]. In the 1990s, LACV emerged in parts of southern Appalachia [11,12] and by the early 2000's, the majority of LACV infections were in West Virginia, North Carolina, and Tennessee [2,13].

Haddow and Odoi (2009) conducted the most recent investigation of the geographic clustering of neuroinvasive LACV disease. Their results demonstrated that from 2003–2007, LACV disease was highly clustered in four focal areas (western North Carolina/ east Tennessee, West Virginia, south-central Ohio, and southwestern Wisconsin), with the highest disease risks in southern Appalachia. Despite its dramatic geographic shift into southern Appalachia, there is evidence that LACV risk is geographically persistent at fine spatial scales [14–16]. If the long-term geographic distribution of LACV disease risk is persistent, public health officials may be able to efficiently reduce disease incidence by focusing resources on areas with persistent risk.

La Crosse virus disease became nationally notifiable in the US in 2003, requiring state health departments to report probable and confirmed human LACV infections to the national arboviral disease surveillance system, ArboNET [17]. As of this writing, 24 years of LACV disease data are available in ArboNET, offering the opportunity to investigate how the geographic distribution of LACV disease risk has changed over time. This study has two objectives: (1) investigate changes in the distribution of county-level geographic clusters of LACV disease from 2003 to 2021 and (2) investigate socioeconomic and demographic predictors of county-level LACV disease risk in states containing persistent high-risk clusters. County-level incidence risks, geographic high-risk clusters, and significant predictors of neuroinvasive LACV disease risk for the time periods of 2003–2007, 2008–2012, 2013–2017, and 2018–2021 are presented. We test the hypotheses that neuroinvasive LACV disease risk clusters are geographically persistent, and that county-level incidence risk can be predicted by socioeconomic and demographic variables.

Methods

Study area and period. This study was conducted in the eastern US, including the entire Appalachian region. The study area consisted of 31 states and 2,046 counties (Fig 1). To compare the geographic distribution of neuroinvasive LACV disease over the study period (2003–2021), data were grouped into the following four time periods for analyses: 2003–2007, 2008–2012, 2013–2017, and 2018–2021. For the investigation of county-level predictors of disease risk, we only included the states that contained high-risk clusters in every time period (Fig 1).

Case Definition. Neuroinvasive LACV disease is reported to ArboNET when patients have symptoms of meningitis, encephalitis, acute flaccid paralysis, or other disfunctions of the central nervous system and evidence of LACV-specific IgM antibodies [18]. Confirmed cases must meet specific laboratory criteria confirming active LACV infection at time of illness. If laboratory criteria for a confirmed case are not met, cases are reported as probable [18].

Case Data. We obtained all probable and confirmed cases of neuroinvasive LACV disease reported in the US from 2003 to 2021 from the US national surveillance system for arboviral diseases, ArboNET. The data were provided at the county level and only included the annual number of cases per county. Demographic information (e.g., age, sex) and epidemiolocal information (e.g., date confirmed) about each case were not available.

County Level Demographic and Socioeconomic Data. We used the National Historical Geographic Information System (NHGIS) to obtain US census data [19]. To account for changes in demography, we matched census data from multiple years with different time periods. We obtained county-level demographic and socioeconomic data from the 2005, 2009, 2015, and 2019 American Community Survey (ACS) and from the 2010 decennial census (Table 1). We chose the following variables as potential predictors based on existing evidence of their association with LACV disease [2,13,20]: percentage of the population with a high school diploma or less, median household income, median year that households were built, population density per square kilometer, housing density per square kilometer, and percentage of the population that is white.

Table 1. Time period-specific data sources for county-level total population and potential predictor variables.

Time Period	Data Source (Total Population)	Data Source (Potential Predictors)
2003-2007	2005 5-year ACS[1]	2009 5-year ACS
2008-2012	2010 Decennial Census	2010 Decennial Census
2013-2017	2015 5-year ACS	2015 5-year ACS
2018-2021	2019 5-year ACS	2019 5-year ACS

[1] American Community Survey

Data Cleaning and Visualization. We used R version 4.0.3 [21] for data cleaning and created maps with ArcGIS Pro version 2.6.0 [22].

Incidence Risk Comparison. We calculated the crude county-level incidence risks for each time period and presented the risks as choropleth maps using Jenk's classification scheme. To investigate temporal changes in risk, we visually assessed crude risk maps and compared mean county-level risks among states. Because states with high risks included many counties that did not report cases, we also calculated mean county-level incidence risks among only the counties that reported neuroinvasive LACV disease.

Geographic Cluster Analysis. We generated county-level queen spatial weights using the R package spdep version 1.2.2. for use in cluster detection [23]. We then used Tango's restricted flexible spatial scan statistic specifying the Poisson model to identify high-risk disease clusters [24] using the R package rflexscan version 1.0.0 [25]. We limited the scanning window size to a maximum of 40 counties and used a stringent significance level of 0.01 to avoid detection of excessively large clusters. To visually assess changes in the distribution of high-risk clusters over time, we mapped

the significant clusters along with their relative risks (RR). We also calculated the number of time periods that each county appeared in a significant high-risk cluster to identify counties that were consistently present in the clusters (minimum = 0 time periods, maximum = 4 time periods).

Predictor Investigation. To avoid problems with multicollinearity, we calculated univariable correlations between the potential predictors using Spearman rank correlations. Only one of a pair of highly correlated ($|r_s| > 0.7$) potential predictors was retained for further assessment. For model fitting, we used the R package glmmTMB version 1.1.2.3 [26]. In all models, the dependent variable was the number of neuroinvasive LACV disease cases per county with the natural log of the county-level population as the offset. Initially we used a Poisson distribution, but overdispersion was consistently detected. Therefore, we used a negative binomial distribution with a log-link. The models included county-level cumulative cases grouped by time period. To account for repeated observations across the four time periods, we included county and time period as random effects with random intercepts.

Prior to fitting multivariable models, we fit univariable models of disease risk with each potential predictor using a critical p-value of 0.10. We included variables that were significantly associated with disease risk in manual backwards elimination with a significance level of $p < 0.05$ to identify the most parsimonious model. To assess confounding, we calculated the change in model coefficients before and after eliminating each variable; if a predictor's coefficient changed by >20%, we considered the eliminated variable a confounder and included it in the final model. Once we

identified the final model, we assessed spatial autocorrelation in the residuals with Moran's I and 9,999 Monte Carlo permutations for significance testing.

Ethical Statement

The University of Tennessee Institutional Review Board (IRB Number: UTK IRB-22-07077-XM) determined that IRB review was not required because this research did not meet the definition of ~~private identifiable~~ information.

Results

Incidence risk comparison. From 2003 to 2021, a total of 1,261 cases of neuroinvasive LACV disease were reported in the study area. The mean number of annual cases declined across the time periods, with an overall average of 66.37 annual cases from 2003–2021 (Table 2). County-level incidence risk for the entire study period ranged from 0 to 126.23, with highest mean county-level incidence risks in West Virginia, North Carolina, Ohio, Tennessee, and Wisconsin. Incidence risks were consistently high in western North Carolina, east Tennessee, West Virginia, and Ohio and intermittently high in southwest Wisconsin (Fig 1, Fig 2). Mean county-level incidence risks declined over time in all the highest-risk states except for Ohio, where risk increased throughout the study period (Table 2). When including only the counties that reported at least one case from 2003–2021, mean county-level incidence risk was substantially higher than when counties not reporting cases were included (Table 2).

Table 2. Descriptive summary of neuroinvasive La Crosse virus disease risks for the entire eastern United States (US) and the four US states with the highest average incidence risks from 2003-2021.

Time period	Mean annual number of cases	Range of county-level incidence risks per 100,000	Mean county-level incidence risk per 100,000 (**West Virginia**)	Mean county-level incidence risk per 100,000 (**North Carolina**)	Mean county-level incidence risk per 100,000 (**Ohio**)	Mean county-level incidence risk per 100,000 (**Tennessee**)	Mean county-level incidence risk per 100,000 (**Wisconsin**)
2003–2007	81.4	0–57.2	4.18	2.06	1.23	1.30	0.78
2008–2012	68.6	0–47.5	3.62	2.01	1.47	0.98	0.19
2013–2017	59.4	0–34.5	1.21	1.76	1.56	1.20	0.49
2018–2021	53.5	0–28.1	0.61	1.21	1.71	0.63	0.04
**2003–2021**	66.37	0–126.23	2.57	1.68	1.49	1.00	0.37
**Only Including Counties that Reported Cases (2003–2021):**			10.65	10.05	3.19	5.59	3.14

Geographic Cluster Analysis. The number, size, and shape of statistically significant high-risk clusters varied across the time periods, but their general geographic distribution was consistent over time (Fig 3). In each period, the primary cluster included counties in western North Carolina and east Tennessee, and the second and third clusters included counties in either West Virginia or Ohio. A high-risk cluster was identified in the upper Midwest from 2003–2007 and 2013–2017, but that region had no clusters in the other time periods. Counties within high-risk clusters had extremely high RRs compared to the whole study area, ranging from 18.02 (2003–2007, Cluster 5) to 70.33 (2003–2007, Cluster 2). The cluster in West Virginia had the highest RR for time periods 2003–2007 and 2008–2012, and the primary cluster spanning western North Carolina and east Tennessee had the highest RR for time periods 2013–2017 and 2018–2021. Overall, across all study periods, 97 counties fell within a high-risk cluster at least once and 74% of these were in the Appalachian region. Of the 97 counties in high-risk clusters, 44 (44.90%) were in a cluster in at least 3 of the time periods, 64 (65.3%) were in a cluster in at least two periods, and 33 (33.67%) were in a cluster in only one period. Importantly, 23 (23.47%) counties were in a high-risk cluster in every time period, and each of those counties were in either North Carolina, Tennessee, West Virginia, or Ohio (Fig 4).

Predictors of County-level LACV Disease Risk. All potential predictors had significant associations with LACV disease risk in univariable analyses (Table 3). In the final model, disease risk was higher in counties where more of the population was white, or the median household income was lower (Table 4). There was no evidence of spatial correlation in the residuals of the final model (Moran's I = -0.02; p = 0.93).

Table 3. Results of univariable analyses for potential predictors of neuroinvasive La Crosse virus disease risk in Ohio, West Virginia, North Carolina, and Tennessee from 2003–2021.

Predictor	Coefficient	95% CI[1]	p
% Population under 20 years	-0.17	-0.32, -0.027	**0.01**
% Population that is White	0.08	0.06, 0.10	**< 0.001**
Median Year Housing Built	-0.05	-0.08, -0.02	**< 0.001**
Median Household Income per 1,000 USD	-0.03	-5.77, -1.18	**0.003**
Population Density (km^2)	-0.003	-0.005, -0.001	**0.002**

[1] Confidence Interval

Table 4. Significant predictors of county-level neuroinvasive La Crosse virus disease risk in Ohio, West Virginia, North Carolina, and Tennessee from 2003–2021.

Predictor	Coefficient (IRR)[1]	95% CI[2]	p
Intercept	-18.27 (1.16)	-20.32, -16.22	**< 0.001**
% Population that is White	0.079 (1.08)	0.059, 0.099	**< 0.001**
Median Household Income per 1,000 USD	-0.030 (0.97)	-0.046, -0.014	**< 0.001**

[1] Incidence Risk Ratio
[2] Confidence Interval

Discussion

Although it is the second-most common cause of arboviral disease in the US, it is tempting to minimize the importance of LACV because of its relatively low annual incidence in comparison to West Nile Virus, which usually causes thousands of annual disease cases [27]. However, it is important to recognize that unlike West Nile virus, which is nationally distributed, the burden of LACV disease consistently falls on populations living in a few high-risk areas [5,13]. Our study demonstrates that the geographic distribution of neuroinvasive LACV disease was consistent from 2003 to 2021. High-risk clusters were present in Appalachia throughout the study period, and relative risks in those clusters were excessively high. Because LACV disease is mostly restricted to small clusters of rural counties, incidence risks in high-risk foci are severely underestimated when measured at the national or state levels [14]. For example, West Virginia had a mean annual state-level incidence risk of 0.61 cases per 100,000 people from 2003–2019 [2]. Comparatively, we found that the mean annual risk among West Virginia counties that reported at least one case from 2003–2021 was nearly 20-times higher. Public health officials should consider the highly focal nature of LACV risk and appropriately allocate resources to reduce the disease burden in high-risk areas.

Haddow and Odoi (2009) identified four distinct geographic areas with high-risk clusters from 2003–2007 (West Virginia, western North Carolina/east Tennessee, Ohio, and Iowa/Wisconsin). We found that those high-risk foci were consistently present from 2003–2021, with the exception of the Iowa/ Wisconsin cluster, which was intermittently present. This finding confirms our hypothesis of geographically persistent high-risk clusters and supports a growing body of evidence that LACV risk is spatially persistent.

For example, from 1966–1995, there was a consistent geographical pattern of LACV disease clustering in three Illinois counties [15]. More recently, three unique instances of spatially-linked noncoincident cases of LACV disease were reported in North Carolina from 2002–2017, with two cases occurring at a single household seven years apart [16]. There are also several examples of LACV-infected mosquitoes being collected at case sites in years following LACV disease diagnoses, further suggesting a persistent household-level exposure [1,28,29]. Taken together with our findings, it is clear that LACV risk depends on geography, and that the distribution of disease is highly predictable. Therefore, LACV transmission may be prevented with targeted mosquito surveillance, mosquito control, and community education in areas with previously documented risk.

While limiting our investigation to states with persistent high-risk clusters (Tennessee, North Carolina, West Virginia, and Ohio), we found that neuroinvasive LACV disease risk increased when county-level income was low or when more of the population was white, confirming our hypothesis that county-level LACV disease risk can be predicted by socioeconomic and demographic factors. The relationships between the predictors and disease risk appears to represent the underlying structure of the high-risk populations. Appalachia is largely comprised of rural, socioeconomically disadvantaged populations that face severe disparities in disease and mortality risk [30–33]. Treatment for neuroinvasive LACV disease treatment can be extremely expensive, especially among children who develop long-term neurologic sequelae [34], so the association of lower median household income with higher disease risks is troubling and should be considered when allocating resources for disease prevention.

The mean annual incidence of neuroinvasive LACV disease declined throughout the study period. We are unaware of any large-scale efforts to prevent LACV disease, so we can only speculate on reasons for that decline. There was a renewed focus on LACV surveillance following its emergence in southern Appalachia in the 1990's [10], but the intensity of testing for LACV infections may have declined in recent years. LACV infections are notoriously underreported, which confounds our understanding of the associated disease burden [3,10,35,36]. It is also possible that the persistent presence of LACV in high-risk foci has resulted in high rates of human immunity or LACV-resistant mosquito populations [36–38]. Another important consideration is the existence of multiple LACV variants, of which only one is known to be pathogenic. Novel LACV variants are sometimes detected outside of high-risk areas, including in New England, where no case of LACV disease has been reported [39]. If non-pathogenic LACV variants exist in high-risk areas, they may affect disease incidence by competing with the pathogenic variant.

There are several important limitations to our study. ArboNET is a passive surveillance system, and data is limited to cases that are both detected and reported. LACV infections are usually asymptomatic, and symptomatic cases can be mistaken as herpes simplex encephalitis or bacterial meningitis [7,9]. If individuals living outside of high-risk areas less likely to be accurately diagnosed with LACV infections, then the distribution of cases presented here will reflect that bias. An additional limitation is that ArboNET does not provide case-specific data at the county level, so we were unable to standardize the cases by age or sex. Therefore, differing demographic structures may confound risk comparisons among counties or states. Finally, it is important to note that

the number of annual LACV disease cases is often small, even when aggregated at the county level. Consequently, small differences in county-level incidence among time periods affected the sizes and shapes of high-risk clusters numbers, especially from 2018–2021, which was one year shorter than the other three periods.

Conclusions

The geographic distribution of neuroinvasive La Crosse virus (LACV) disease remained consistent from 2003–2021, with the geographic clusters consistently present in in West Virginia, western North Carolina, east Tennessee, and Ohio. County-level risk for neuroinvasive LACV disease in those states increased when more of the population was white or when median household income was lower. These findings add to the existing body of research indicating that LACV disease is spatially persistent in the Appalachian region. Due to the persistent focus of neuroinvasive LACV disease in disadvantaged communities, allocation of resources for targeted intervention is warranted.

Data Availability

The Centers for Disease Control Division of Vector-Borne Diseases approved the release of a summarized dataset containing cases aggregated across time periods, which will be sufficient for replication of all analyses in this manuscript. That dataset will be submitted to reviewers and published in a public data repository upon acceptance. The authors do not have legal authority to release the per-year disease dataset but interested parties can request it by submitting an email request to the at dvbid2@cdc.gov.

References

1. Westby KM, Fritzen C, Paulsen D, Poindexter S, Moncayo AC. La Crosse encephalitis virus infection in field-collected *Aedes albopictus*, *Aedes japonicus*, and *Aedes triseriatus* in Tennessee. J Am Mosq Control Assoc. 2015;31: 233–241. doi:10.2987/moco-31-03-233-241.1

2. Vahey GM, Lindsey NP, Staples JE, Hills SL. La Crosse virus disease in the United States, 2003–2019. Am J Trop Med. 2021;105: 807–812. doi:10.4269/ajtmh.21-0294

3. Kappus KD, Monath TP, Kaminski RM, Calisher CH. Reported encephalitis associated with California serogroup virus infections in the United States, 1963-1981. Prog Clin Biol Res. 1983;123: 31–41.

4. Leisnham PT, Juliano SA. Impacts of climate, land use, and biological invasion on the ecology of immature *Aedes* mosquitoes: implications for La Crosse emergence. EcoHealth. 2012;9: 217–228. doi:10.1007/s10393-012-0773-7

5. Gaensbauer JT, Lindsey NP, Messacar K, Staples JE, Fischer M. Neuroinvasive arboviral disease in the United States: 2003 to 2012. Pediatrics. 2014;134: e642–e650. doi:10.1542/peds.2014-0498

6. Thompson WH, Kalfayan B, Anslow RO. Isolation of California encephalitis group virus from a fatal human illness. Am J Epidemiol. 1965;81: 245–253. doi:10.1093/oxfordjournals.aje.a120512

7. McJunkin JE, De Los Reyes EC, Irazuzta JE, Caceres MJ, Khan RR, Minnich LL, et al. La Crosse encephalitis in children. N Engl J Med. 2001;344: 801–807. doi:10.1056/NEJM200103153441103

8. Boutzoukas AE, Freedman DA, Koterba C, Hunt GW, Mack K, Cass J, et al. La Crosse virus neuroinvasive disease in children: a contemporary analysis of clinical/neurobehavioral outcomes and predictors of disease severity. Clin Infect Dis. 2022; ciac403. doi:10.1093/cid/ciac403

9. Miller A, Carchman R, Long R, Denslow SA. La Crosse viral infection in hospitalized pediatric patients in Western North Carolina. Hosp Pediatr. 2012;2: 235–242. doi:10.1542/hpeds.2012-0022

10. Jones TF, Craig AS, Nasci RS, Patterson LER, Erwin PC, Gerhardt RR, et al. Newly recognized focus of La Crosse encephalitis in Tennessee. Clin Infect Dis. 1999;28: 93–97. doi:10.1086/515087

11. Erwin PC, Jones TF, Gerhardt RR, Halford SK, Smith AB, Patterson LER, et al. La Crosse Encephalitis in eastern Tennessee: clinical, environmental, and entomological characteristics from a blinded cohort study. Am J Epidemiol. 2002;155: 1060–1065. doi:10.1093/aje/155.11.1060

12. Jones TF, Erwin PC, Craig AS, Baker P, Touhey KE, Patterson LER, et al. Serological survey and active surveillance for La Crosse virus infections among children in Tennessee. Clin Infect Dis. 2000;31: 1284–1287. doi:10.1086/317458

13. Haddow AD, Odoi A. The incidence risk, clustering, and clinical presentation of La Crosse virus infections in the eastern United States, 2003–2007. Rénia L, editor. PLoS ONE. 2009;4: e6145. doi:10.1371/journal.pone.0006145

14. Haddow AD, Jones CJ, Odoi A. Assessing risk in focal arboviral infections: are we missing the big or little picture? Jones JH, editor. PLOS ONE. 2009;4: e6954. doi:10.1371/journal.pone.0006954

15. Kitron U, Michael J, Swanson J, Haramis L. Spatial analysis of the distribution of Lacrosse encephalitis in Illinois, using a geographic information system and local and global spatial statistics. Am J Trop Med Hyg. 1997;57: 469–475. doi:10.4269/ajtmh.1997.57.469

16. Byrd BD, Williams CJ, Staples EJ, Burkhalter KL, Savage HM, Doyle MS. Spatially associated coincident and noncoincident cases of La Crosse encephalitis - North Carolina, 2002-2017. MMWR Morb Mortal Wkly Rep. 2018;67: 1104–1105.

17. Centers for Disease Control and Prevention. ArboNET disease maps. 2022 [cited 18 May 2022]. Available: https://wwwn.cdc.gov/arbonet/maps/ADB_Diseases_Map/index.html

18. Centers for Disease Control and Prevention. Arboviral diseases, neuroinvasive and non-neuroinvasive 2015 case definition. 2015 [cited 22 Apr 2022]. Available: https://ndc.services.cdc.gov/case-definitions/arboviral-diseases-neuroinvasive-and-non-neuroinvasive-2015/

19. Manson S, Schroeder J, Van Riper D, Kugler T, Ruggles S. IPUMS National Historical Geographic Information System: Version 16.0 [dataset]. In: nhgis.org [Internet]. 2022. Available: nhgis.org

20. Haddow AD, Bixler D, Schuh AJ. The demographic and socioeconomic factors predictive for populations at high-risk for La Crosse virus infection in West Virginia. Braga EM, editor. PLOS ONE. 2011;6: e25739. doi:10.1371/journal.pone.0025739

21. R Core Team. R: A language and environment for statistical computing. Vienna, Austria: R Foundation for Statistical Computing; 2020. Available: https://www.R-project.org/

22. Esri Inc. ArcGIS Pro (Version 2.6.0). Esri Inc. 2020. Available: https://www.esri.com/en-us/arcgis/products/arcgis-pro/overview

23. Bivand R. R packages for analyzing spatial data: a comparative case study with areal data. Geog Anal. 2022;n/a. doi:10.1111/gean.12319

24. Tango T, Takahashi K. A flexible spatial scan statistic with a restricted likelihood ratio for detecting disease clusters. Stat Med. 2012;31: 4207–4218. doi:10.1002/sim.5478

25. Otani T, Takahashi K. rflexscan: the flexible spatial scan statistic. 2021. Available: https://CRAN.R-project.org/package=rflexscan

26. Brooks ME, Kristensen K, Benthem KJ van, Magnusson A, Berg CW, Nielsen A, et al. glmmTMB balances speed and flexibility among packages for zero-inflated generalized linear mixed modeling. The R Journal. 2017;9: 378–400.

27. Centers for Diseaes Control and Prevention. Final cumulative maps and data | West Nile virus. 5 Jan 2022 [cited 3 Aug 2022]. Available: https://www.cdc.gov/westnile/statsmaps/cumMapsData.html

28. Cook C, Blesi A, Brozak S, Lenhart S, Reed H, Urquhart C, et al. La Crosse virus spread within the mosquito population in Knox County, TN. PLOS ONE. 2021;16: e0249811. doi:10.1371/journal.pone.0249811

29. Gerhardt RR, Gottfried KL, Apperson CS, Davis BS, Erwin PC, Smith AB, et al. First isolation of La Crosse virus from naturally infected *Aedes albopictus*. Emerg Infect Dis. 2001;7: 807–11. doi:10.3201/eid0705.017506

30. Marshall JL, Thomas L, Lane NM, Holmes M, Arcury TA, Randolph R, et al. Health disparities in Appalachia. PDA, Inc; Cecil G. Sheps Center for Health Services Research; Appalachian Regional Commission; 2017. Available: https://www.arc.gov/wp-content/uploads/2020/06/Health_Disparities_in_Appalachia_August_2017.pdf

31. Appalachian Regional Commission. Population and age in Appalachia. 2019 [cited 9 May 2022]. Available: https://www.arc.gov/appalachias-population/

32. Hotez PJ. Neglected Infections of poverty in the United States of America. PLOS Negl Trop Dis. 2008;2: e256. doi:10.1371/journal.pntd.0000256

33. Murray CJL, Kulkarni S, Ezzati M. Eight Americas: new perspectives on U.S. health disparities. Am J Prev Med. 2005;29: 4–10. doi:10.1016/j.amepre.2005.07.031

34. Utz JT, Apperson CS, Maccormack JN, Salyers M, Dietz EJ, Mcpherson JT. Economic and social impacts of La Crosse encephalitis in western North Carolina. American Journal of Tropical Medicine and Hygiene. 2003;69: 509–518. doi:https://doi.org/10.4269/ajtmh.2003.69.509

35. Rust RS, Thompson WH, Matthews CG, Beaty BJ, Chun RW. La Crosse and other forms of California encephalitis. J Child Neurol. 1999;14: 1–14. doi:10.1177/088307389901400101

36. Szumlas DE, Apperson CS, Hartig PC, Francy DB, Karabatsos N. Seroepidemiology of La Crosse virus infection in humans in western North Carolina. Am J Trop Med Hyg. 1996;54: 332–337. doi:10.4269/ajtmh.1996.54.332

37. Costanzo KS, Muturi EJ, Montgomery AV, Alto BW. Effect of oral infection of La Crosse virus on survival and fecundity of native *Ochlerotatus triseriatus* and invasive *Stegomyia albopicta*: effect of oral infection of La Crosse virus. Med Vet Entomol. 2014;28: 77–84. doi:10.1111/mve.12018

38. Grimstad PR, Craig GB, Ross QE, Yuill TM. *Aedes triseriatus* and La Crosse virus: geographic variation in vector susceptibility and ability to transmit. Am J Trop Med Hyg. 1977;26: 990–996. doi:10.4269/ajtmh.1977.26.990

39. Eastwood G, Shepard JJ, Misencik MJ, Andreadis TG, Armstrong PM. Local persistence of novel regional variants of La Crosse virus in the Northeast USA. Parasit Vectors. 2020;13: 569. doi:10.1186/s13071-020-04440-4

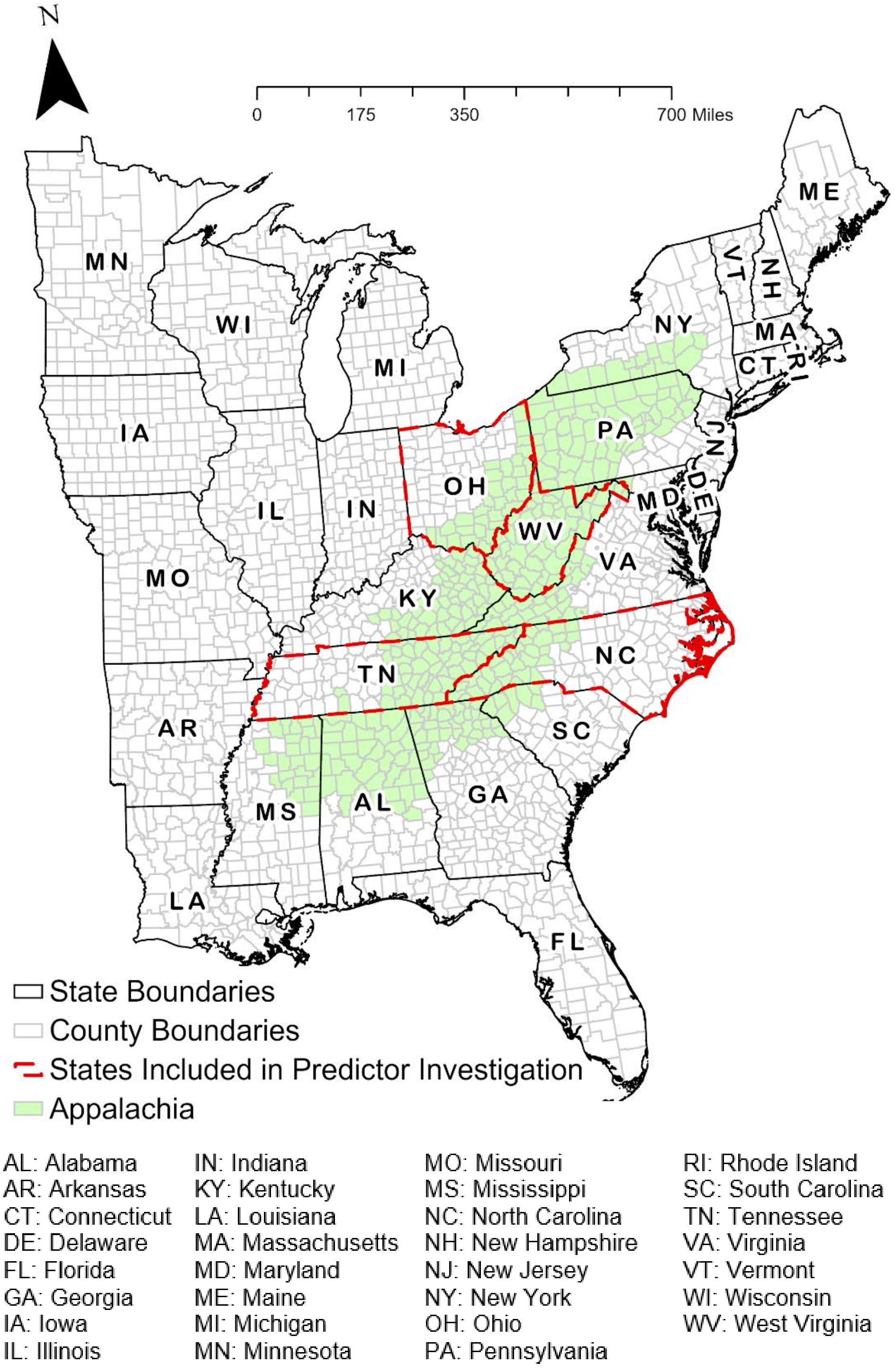

Fig 1. Map of the study area (eastern United States) with state boundaries, county boundaries, boundaries of states used for investigation of risk predictors, and state abbreviations.

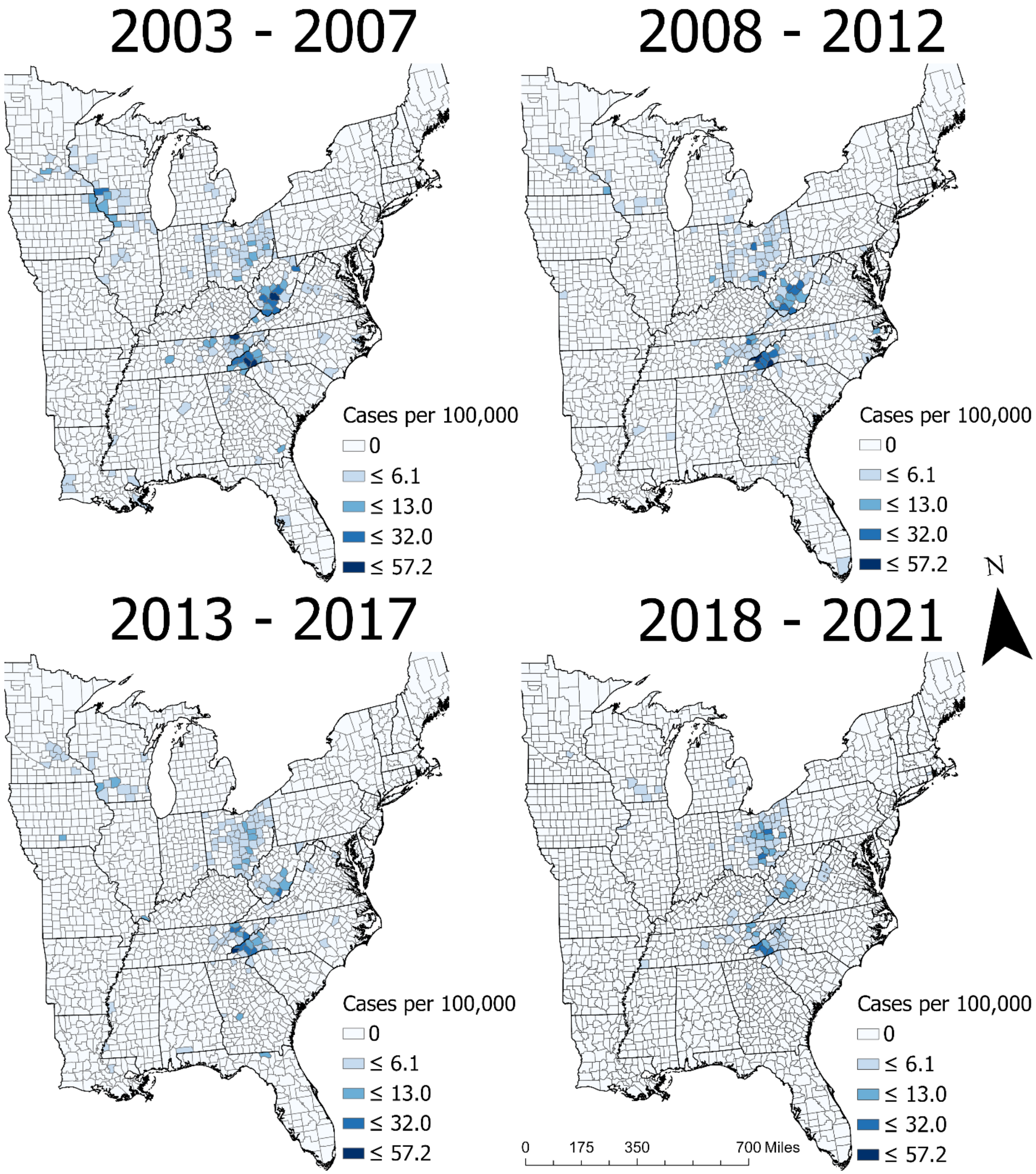

Fig 2. Crude county-level incidence risks of neuroinvasive La Crosse virus disease for the total population in the eastern United States from 2003-2021 (data obtained from ArboNET).

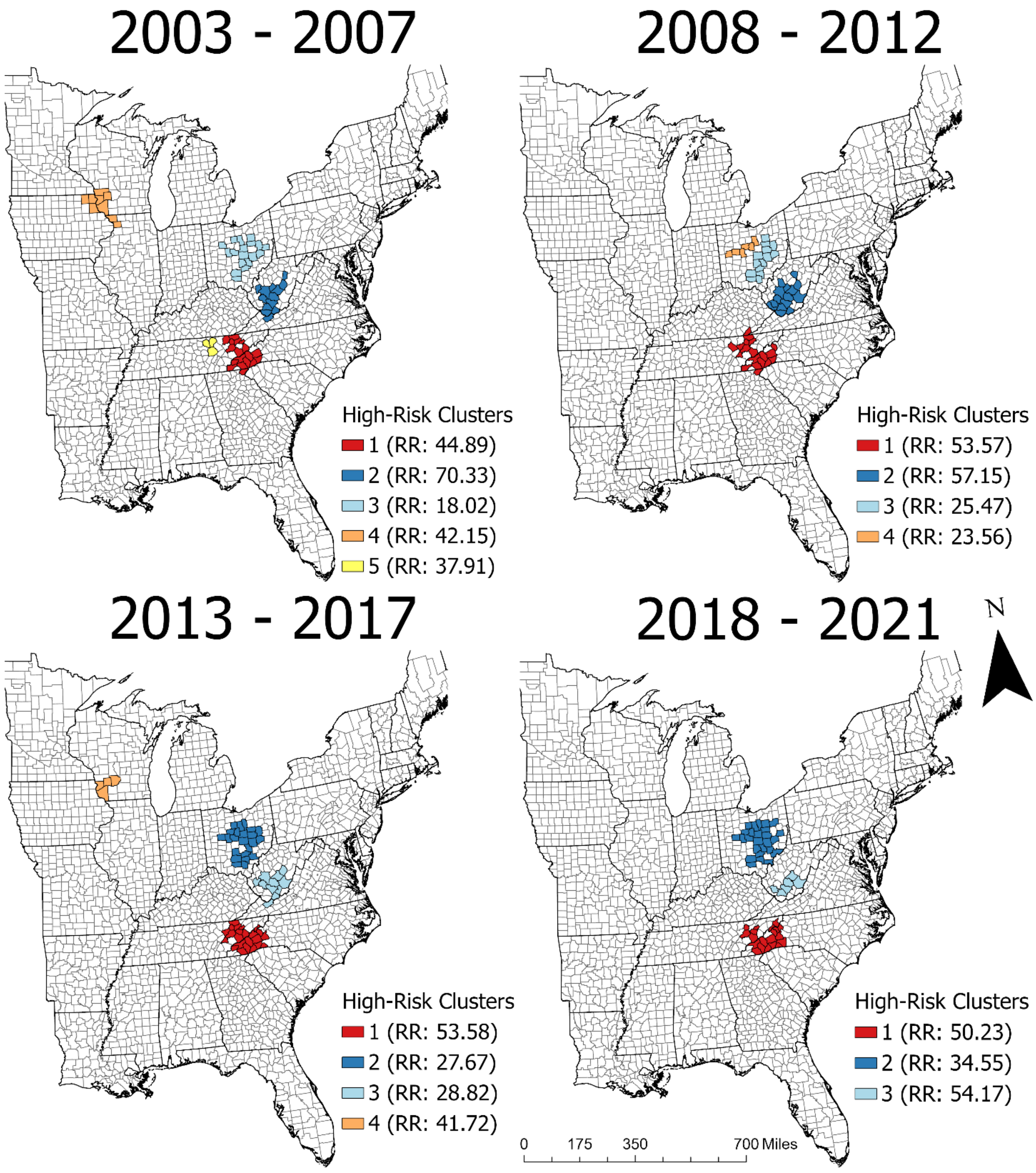

Fig 3. Flexibly shaped high-risk clusters of neuroinvasive La Crosse virus disease for the total population in the eastern United States from 2003-2021 (data obtained from ArboNET).

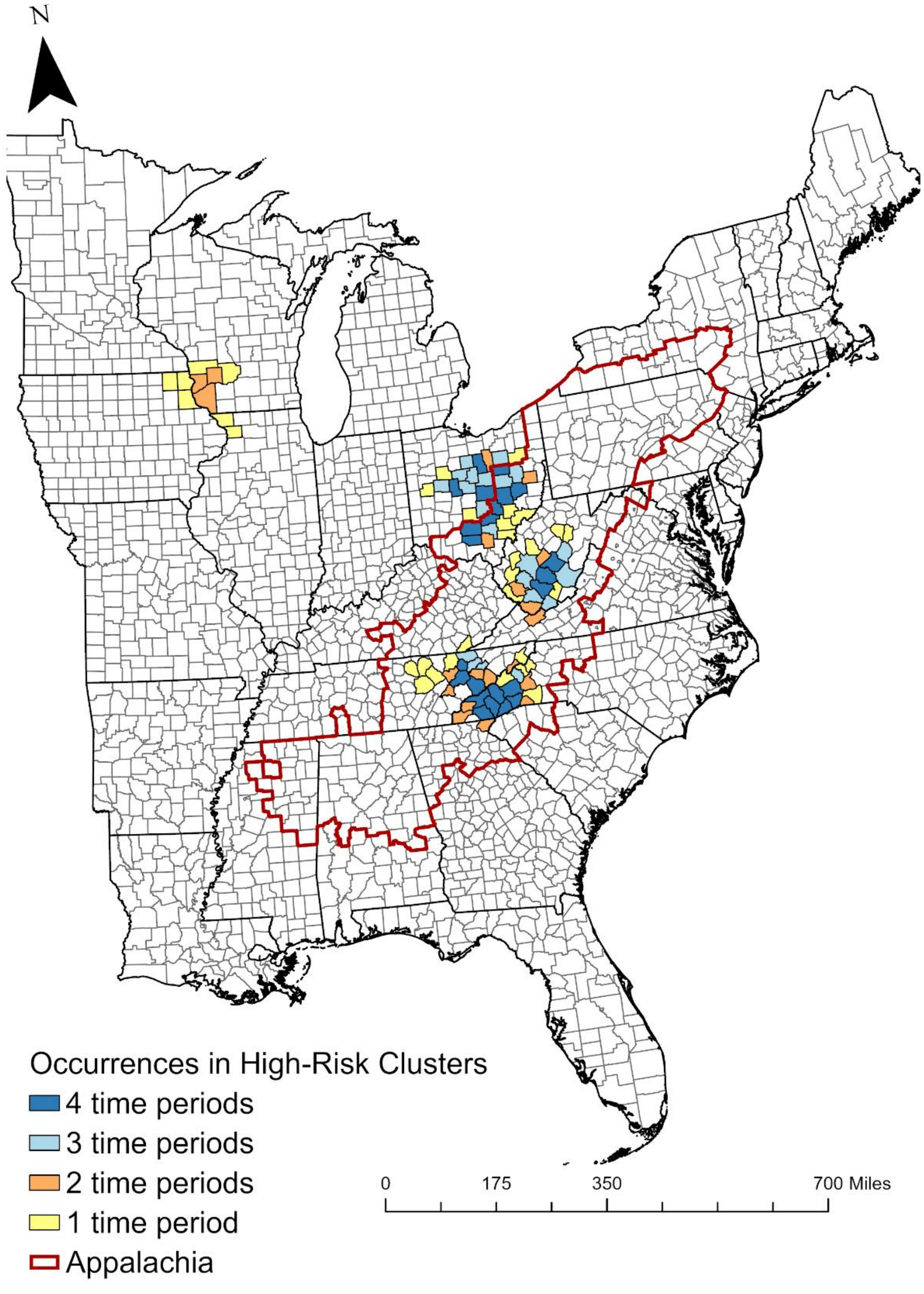

Fig 4. The number of time periods in which each county fell within a high-risk cluster of neuroinvasive La Crosse virus disease from 2003-2021 in the eastern United States (data obtained from ArboNET).

499

Figure 1

Click here to access/download;Figure;Fig 1 - study area.tif

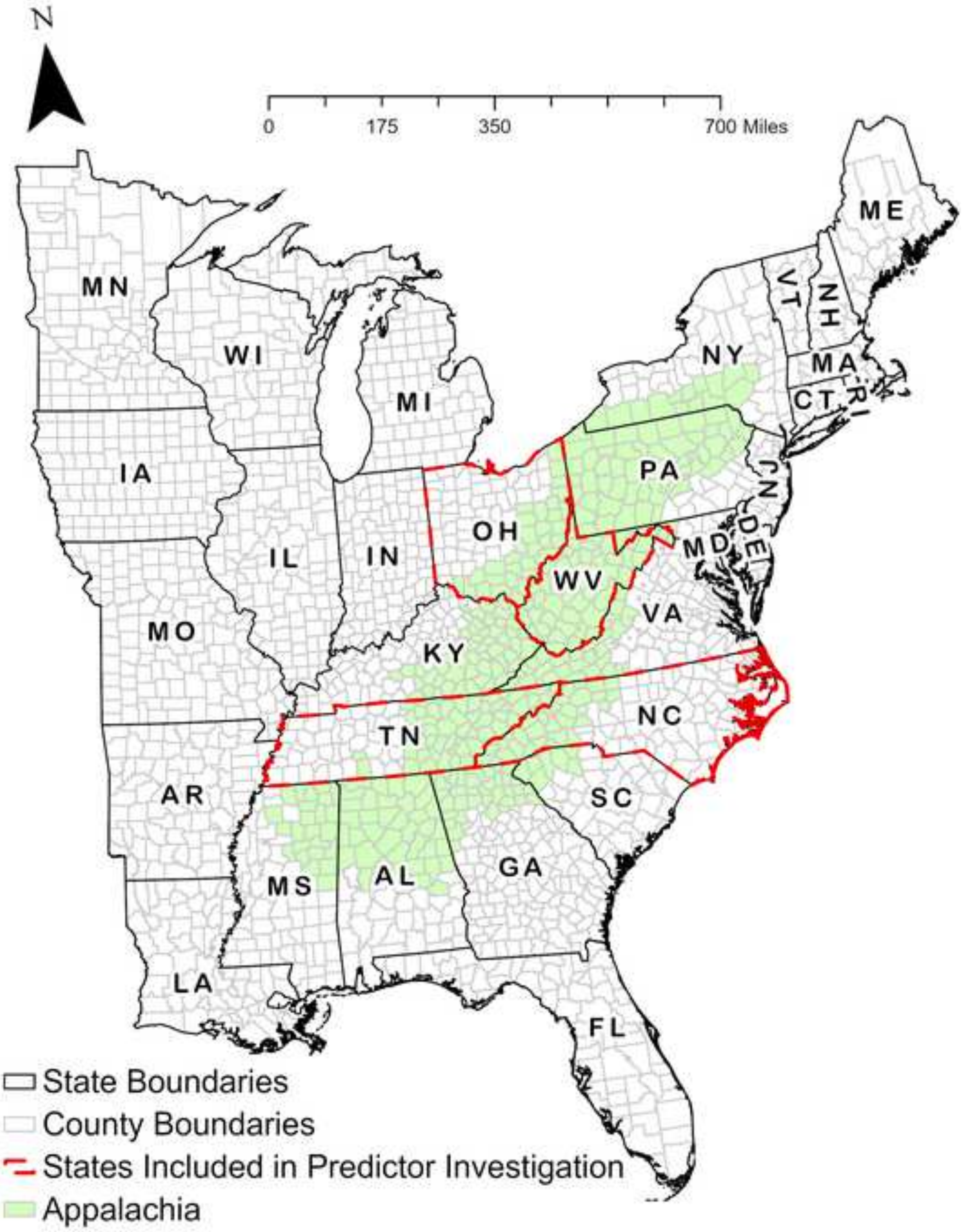

Figure 2

Click here to access/download;Figure;Fig 2 - Risk maps.tif

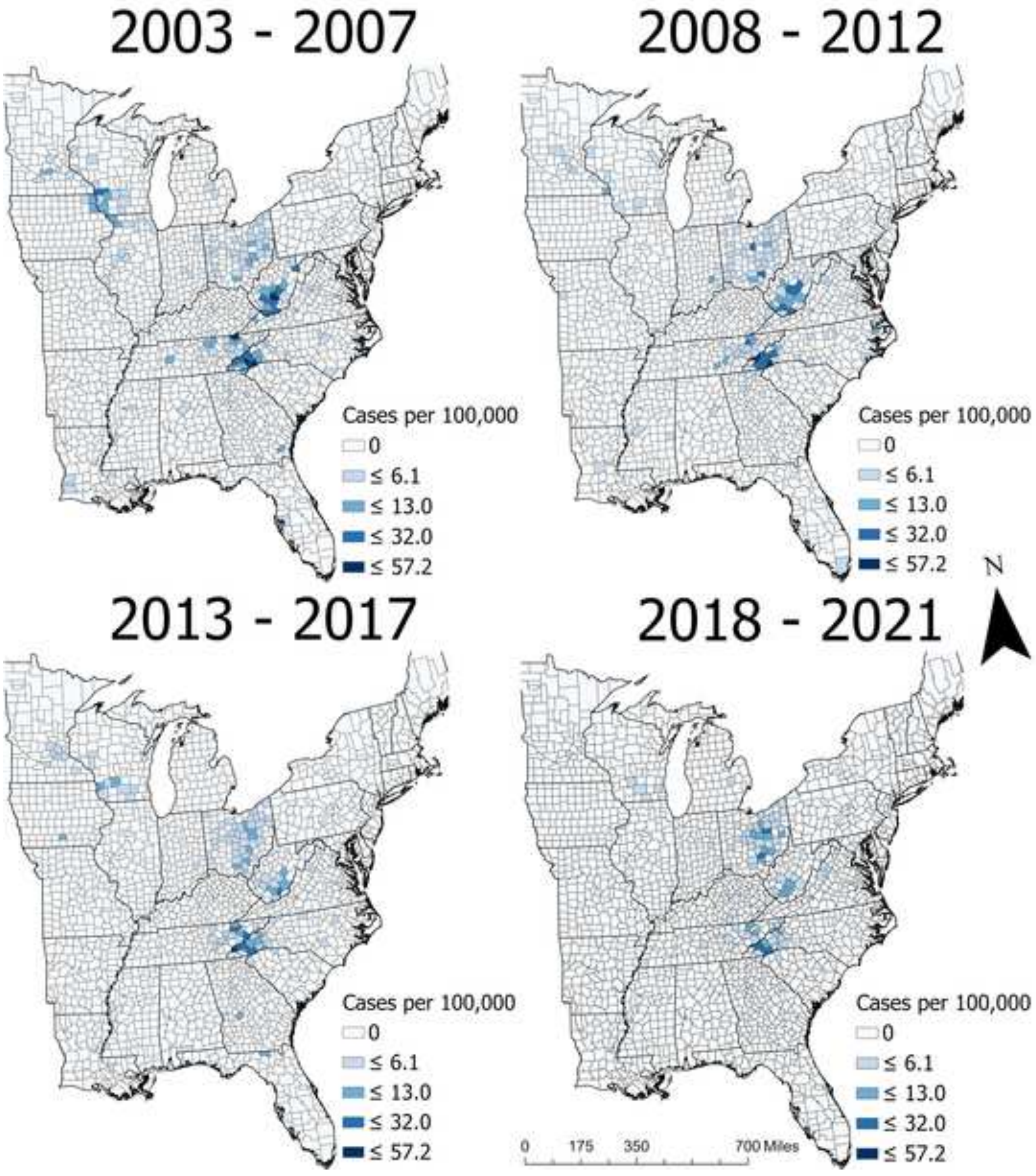

Figure 3

Click here to access/download;Figure;Fig 3 - cluster maps.tif

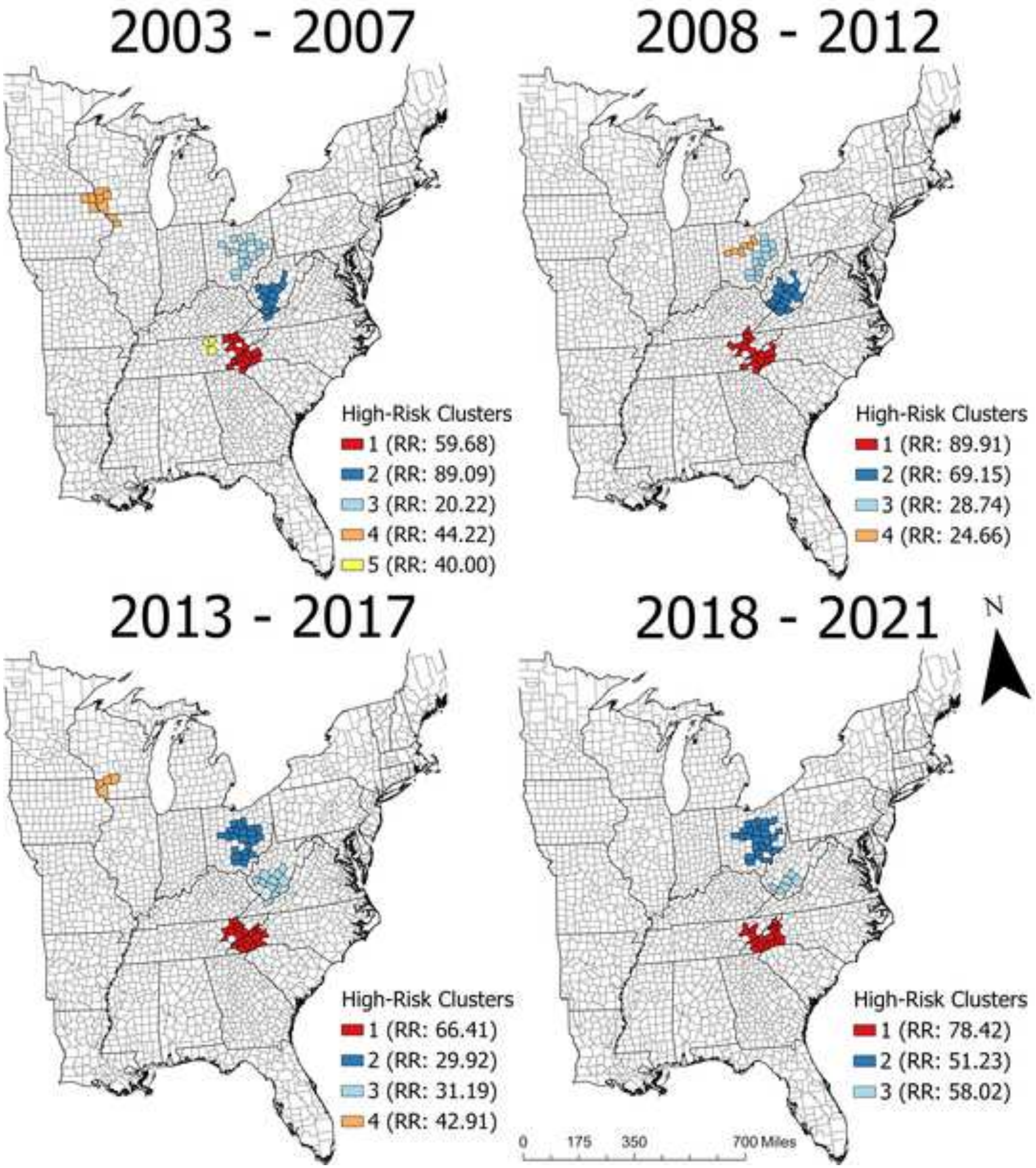

Figure 4

Click here to access/download;Figure;Fig 4 -number of clusters.tif

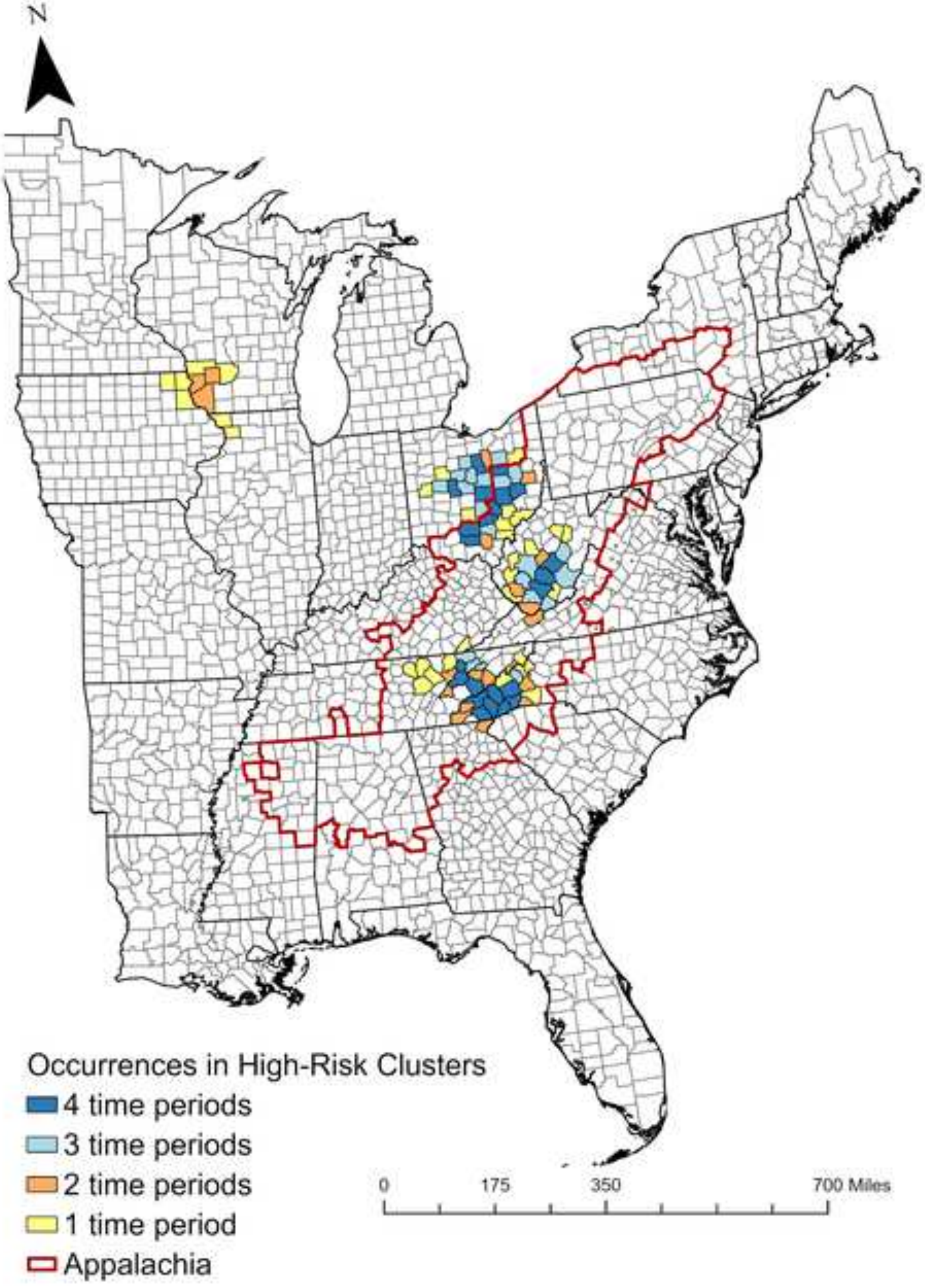

This is an email chain with the CDC Division of Vector-Borne diseases where approval to share the supplementary data file was

Click here to access/download
Supporting Information
ArboNET data sharing approval.pdf

Outcome letter from the University of Tennessee IRB

Click here to access/download
Supporting Information
Outcome_Letter.pdf

Data file with dictionary and necessary data to reproduce analyses.

Click here to access/download

Supporting Information

LAC Data for Publication.xlsx
