# Supplementary material for: Geographically persistent clusters of La Crosse virus disease in the Appalachian region of the United States from 2003 to 2021
Source: PLoS Negl Trop Dis. 2023 Jan 19;17(1):e0011065. doi: 10.1371/journal.pntd.0011065 (PMC9888679; doi:10.1371/journal.pntd.0011065)
Supplement: Attachment — Submitted filename: PNTD-D-22-01013_gv.docx [file pntd.0011065.s002.docx]

In the manuscript titled “Persistent high-risk geographic clusters of neuroinvasive La Crosse virus disease in the socioeconomically disadvantaged Appalachian region of the United States” the authors use descriptive statistics and spatial analysis to describe spatial distribution of neuroinvasive La Crosse virus disease cases over time. They calculated cumulative incidence by county for 4 time periods during 2003–2021. They found that the counties with the highest cumulative incidence were similar across all 4 time periods. They also use a generalized linear mixed model to identify characteristics associated with increased neuroinvasive LACV disease incidence at the county level. They identified that increased county-level neuroinvasive LACV disease incidence was associated with increased proportion of residents being White and decreased median household income. They conclude that geographic distribution of neuroinvasive LACV disease was consistently highest in 4 Appalachian states over 2003–2021 and advocate for prevention resource allocation to be targeted based on socioeconomic disadvantage. In general, the methods seem sound and the findings would be valuable, but there are issues with the presentation of the findings and the manuscript is not clearly or concisely written. The use of maps to communicate findings is very well done, however.

**Title**

The title could be more concise. Consider removing “socioeconomically disadvantaged” from the title, I think it takes away from the main message. I also recommend adding the time frame to the title.

**Abstract**

Line 19: it should be noted that the authors are discussing *reported* LACV disease which may be different than the actual distribution of disease. Changes in awareness, diagnostic capability, and reporting practices could also contribute to the changes in reported disease geographic distribution.

Line 29: I disagree with the interpretation starting in this line. The analysis found association with higher incidence at the county level with increased proportions of White residents and lower median household income, not “high disease risk.” Please clarify the interpretation here.

**Author Summary**

Line 38: this statement should be amended to “pathogen that can cause neuroinvasive disease” as LACV infection can also be asymptomatic or cause febrile illness.

Line 42: again, should be “reported LACV disease”

Line 47: “high-risk clusters of neuroinvasive LACV disease risk” doesn’t really make sense. I believe if you remove the second “risk” it would make sense.

Line 50: same feedback as line 29. The authors seem to be over-interpreting these results.

Line 52: please specify the time period you are referring to, as you’ve also stated that geographic distribution changed in the 1990s.

**Introduction**

Line 61: I feel that it is important to also note that LACV infection can be asymptomatic (I believe the authors mention this in the discussion) and can also cause febrile illness. Also, the word “pediatric” is unnecessary and misleading here, I recommend removing it.

Line 63: again, I recommend specifying “reported” LACV disease cases.

Line 67: I’m not sure if this statement is factual, as Ohio has consistently had some of the highest reported case numbers for this time period.

Line 68: recommend specifying nationwide geographic clustering here, as Haddow et al. have another paper from 2011 with spatial analysis of LACV restricted to WV.

Line 72: I know that Appalachia is depicted in the Figure 1 map, but it might be good to define where that region is earlier in the manuscript, especially given the international audience of PLOS Neglected Tropical Diseases.

Line 79: I recommend deleting the clause from “As of this writing, …available in ArboNET” as it is misleading. You’ve just stated that LACV became notifiable in 2003, you are presenting data for 2003-2021, and we can’t really compare pre and post required reporting data periods.

Line 87: I would take out the individual time periods here since you will be presenting them in the methods section. Additionally, I don’t think you present your predictors analysis by those individual time periods.

Line 89: this occurs throughout the manuscript, but I believe this is the first instance. “Incidence risk” is a bit of a redundant term that isn’t used consistently in epidemiology. I believe the authors mean cumulative incidence which is a much more widely used term. I recommend changing “incidence risk” to “cumulative incidence” throughout. Also, the authors refer to their GLMM analysis as “predictive” throughout – can they clarify if this is really meant to be predictive modeling? Predictive feels like a strong conclusion to make from this analysis.

**Methods**

Line 92: can the authors clarify why they restricted to these 31 Eastern US states?

Line 97: can the authors clarify why only the 4 states with consistent high risk clusters were included in this analysis? What is the methodological reason behind excluding those other states?

Line 98: I recommend that the authors put in parentheses in this sentence where the definition of high-risk clusters can be found in the methods? This term has been used multiple times without a definition and it may be unclear for readers who are unfamiliar with spatial analytic methods.

Line 99: looking at the ArboNET case definitions this is a really oversimplified, recommend adding in more detail about the laboratory criteria specifically.

Line 112: I don’t understand the sentence beginning in line 112 “To account for…time periods.” Can the authors re-write this sentence?

Lines 116 – 121: this should be in the methods section titled “Predictor Investigation” as these are the variables included in those models. Additionally, table 3 also lists age as a predictor variable, please ensure that all variables from models are also in methods.

Table 1: why was the 2009 ACS used for the 2003-2007 time period predictors?

Line 129: I question the validity of comparing mean county-level incidence over time or between states. Mean county incidence will be affected by how focal reported disease is in that state (i.e., every county that reports zero cases will dramatically skew the mean). For comparing over time and between states, I recommend calculating cumulative incidence for the entire state for each time period. County-level incidence is appropriate for maps such as Figure 2 and calculating spatial statistics. Additionally, in line 129 I believe the authors mean “among states across time periods.”

Line 133: Can the authors add in a sentence or two with a more lay-person’s description of what these analyses results mean? Something like “to identify high-risk clusters – that is, groups of counties that have a much higher cumulative incidence of disease than the rest of the study area.” Many people will not be familiar with geospatial analytic methods and will be unable to understand your results without some more basic explanation.

Line 140: please list what was used as the comparison group for calculation of relative risk.

Line 146: please list in results whether any variables were excluded because of this collinearity analysis.

Line 148: for the dependent variable please specify whether this was the incidence for the entire study period.

**Results**

Line 175: please give units for incidence (likely per 100,000 population). Additionally, it would be helpful to give the cumulative incidence for the entire study area for this time period so readers have some context for how high the incidence is in WV, NC, TN, OH, and WI. Lastly, I think it would be really helpful to include the cumulative incidence for each state mentioned here in parentheses so readers don’t have to reference the table.

Line 178: It doesn’t look like this information is included in Figure 1 as it says here. Please double check.

Line 180: Strongly recommend deleting the sentence “When including only the counties…included (Table 2).” This isn’t really a finding, it is just a mathematical fact that when you take 0’s out the mean will increase substantially. If the authors want to communicate how focal reported cases were in each state, I would recommend including the number and proportion of counties in each state that reported cases in the study period. I think that is a clearer way to communicate this concept.

Table 2: Again, I advise against using mean county-level incidence and instead recommend calculating incidence for the entire state for each time period. The column names could also be cleaner.

Line 190: I believe the authors should remove “Counties with” here as the RRs are associated with the cluster, and not the counties.

Line 196-201: the way these results are presented is confusing. The authors should consider reporting how many clusters were in each number of time periods – the use of “at least X periods” for some of them is confusing.

Line 204: I strongly recommend including the estimates for measure of effect in this paragraph, along with an interpretation (e.g., a $1000 decrease in median income in a county is associated with an X% increase in neuroinvasive LACV cases in the county). From the manuscript as currently written it is unclear what the magnitude of association/effect is, and the IRRs, while significant, are close to one.

**Discussion**

Line 223: this comparison should not be made. Firstly because one is incidence at the state level and one is mean county incidence in the state. Secondly, the mean county incidence presented here only includes counties that reported a case. This is not a valid comparison.

Line 229: can the authors discuss how the high-risk clusters differed over time? In the results it is mentioned that clusters varied in size, shape, and that the clusters in Iowa/Wisconsin was only present in some of the analyzed time periods. Are there any possible explanations for that?

Line 239: I think it is very important for the authors to also discuss that geographic distribution of LACV disease is highly dependent on distribution of vectors and amplifying hosts. These are complex factors that aren’t mentioned in this manuscript at all. Also, the authors mention here that distribution is highly predictable, but earlier mentioned the expansion into the Southeast in the 1990s. This is a contradictory statement – potentially the authors mean that geographic distribution has been stable for this time period? I think that’s as far as we can conclude based on this data.

Line 244: why was the GLMM analysis restricted to those 4 states? Is there some thought that there might be different factors associated with LACV risk in other states? Also, in this section the authors make too strong of conclusions based on their findings – I think it’s fair to say that there was an association between the two variables and case numbers at the county level, but maybe not that risk can be predicted by these two variables.

Line 252: the word “treatment” is included in this sentence twice

Line 270-282: the limitations paragraph is unclearly written. For example, in the second sentence there are two facts listed but it is unclear how these facts could affect the validity of these findings. The third sentence I believe is alluding to bias from awareness, but it is unclear. Also, there are some limitations that are not addressed: only variables previously associated to be associated with increased LACV risk were included in the GLMM model, so some risk factors might be missed, the GLMM model only included 4 states, so those findings are not generalizable beyond those states, and the temporal analysis did not use statistical methods, so temporal analysis is based on observation and descriptive statistics.

Line 276: please clarify – ArboNET does collect demographic data for cases, but it was just not available in the dataset used for this analysis.

**Conclusions:**

Line 290: please specify that it is persistent in Appalachia since the 1990s. Also recommend adding “economically” in front of disadvantaged communities since your findings only found White race and lower median household income to be associated with county level risk.

Comments to the Editor

I have limited knowledge of the spatial analysis methods used by these authors so I am unable to comment on the validity of these methods or the strict interpretation of those results.
