# Supplementary material for: Geographically persistent clusters of La Crosse virus disease in the Appalachian region of the United States from 2003 to 2021
Source: PLoS Negl Trop Dis. 2023 Jan 19;17(1):e0011065. doi: 10.1371/journal.pntd.0011065 (PMC9888679; doi:10.1371/journal.pntd.0011065)
Supplement: Attachment — Submitted filename: Response to Reviewers.docx [file pntd.0011065.s003.docx]

**Summary of Changes:**

In response to reviewer comments, we made substantial revisions to the submitted manuscript. The changes are mostly aimed at clarifying the methods and results for the reader, and the underlying approach and statistical analyses remain unchanged. We are including updated figures, where the headings now include the “en dash” instead of a hyphen (e.g., 2003 – 2007 instead of 2003 - 2007), but the figures are otherwise unchanged.

We changed some verbiage to maintain consistency with the epidemiological literature. First, we refer to our significant risk predictors as “associated” instead of “predictive”. We also opted to use the term “cumulative incidence” instead of “incidence risk”. We added more detailed definitions of cumulative incidence, risk ratio, and risk clusters in the methods section. Additionally, we clarified our regression approach and offered a more detailed explanation of the cluster analysis that we used. Finally, we changed the term “Incidence Risk Ratio” to “Risk Ratio” in the regression tables; the interpretation is unchanged.

In the results, we revised Table 2 to include statewide cumulative incidence instead of mean county incidence. We also added a new table (Table 3 in the revised manuscript) that includes details of the cluster analyses, including the cumulative incidence for each cluster. This change allows us to clearly compare statewide cumulative incidence to the cumulative incidence within the clusters, with the goal of highlighting the very focal nature of this disease within the states that have disease clusters.

Beyond the changes summarized above, we made many additional edits to the writing that we hope will further clarify our findings, the relevance of those findings, and this study’s limitations.

Below are our direct responses to the reviewers’ comments. **Note that *reviewer comments are italicized* while our responses are not.**

**Reviewer 1:**

**Methods**

*Objectives are clearly stated, study design is appropriate, and there are no ethical concerns with the data*

Thank you.

**Results**

*Analyses are appropriate, results and figures are clearly presented.*

Thank you.

**Conclusions**

*Conclusions are appropriate, limitations are noted, and public health relevance is indicated. While noting that Appalachia is largely comprised of rural, socioeconomically disadvantaged populations that face severe disparities in disease and mortality risk, do you have any thoughts as to why those of lower median household income are at higher risk for LACV disease specifically? Is there any reason there would be higher mosquito breeding? In regards to higher risk in counties with a higher population proportion of white persons, could this be related to better access to health care?*

Thank you for these comments and questions. We added text to the discussion to elaborate on these points to clarify them for other readers. Any explanation for these associations will be largely speculative given the lack of research that adequately explains the distribution of this disease. Because the distribution of LACV disease is clustered in these areas of Appalachia, but rarely reported elsewhere, it seems likely that the distribution of disease risk is a product of some environmental factors associated with Appalachia. If that is correct, then the association of lower income and higher proportion of white persons with disease risk is probably coincidental. In other words, high LACV disease risks in Appalachian counties may be a product of environmental factors, but because Appalachian counties have lower household incomes, low household income would still be statistically associated with risk.

Notably, a household-level study could conceivably identify an association between income and mosquito abundance, which may also be associated with LACV disease risk. In our study, it is not feasible to draw such a conclusion.

**Editorial and Data Presentation Modifications?**

*Consider reporting data to one decimal point only to allow for easier reading
-       Line 103: It is not clear that “probable” cases require laboratory testing, albeit having criteria for notification that are not as strict as those for confirmed cases. Please consider rewording the statement that “laboratory criteria for a confirmed case are not met, cases are reported as probable”*

Thank you for pointing out the misleading nature of the text in this section. We revised the writing to further clarify the definitions of confirmed and probable cases.

*-       Line 213: no capital needed for virus in “West Nile virus”*

We corrected this error.

*-       Line 219: Can abbreviate “relative risks” as previously done*

We abbreviated relative risks to RRs.

*-       Line: 261: Perhaps consider a more scientific word than “notoriously”*

We removed this sentence.

*-       Line 288: Do you mean or “increased” or “was higher” in the sentence “County-level risk for neuroinvasive LACV disease in those states increased when more of the population was white or when median household income was lower”.*

We changed our wording in this sentence.

**Summary and General Comments**

*This is an interesting, nicely written, and well referenced manuscript, and appropriate limitations are noted. The findings are important in terms of considering public health approaches to control.*

Thank you.

**Reviewer 2:**

**Objectives**

*The methods were acceptable; however, two more novel computational approaches exist that the reviewer encourages the author to try on their dataset. Given the limited dataset available, it is unknown if these statistical approaches hold merit. However, the author is requested to attempt them. Details can be found in the attached pdf document.*

Thank you for the suggestions. Regarding a formal statistical test of temporal changes in cumulative incidence: we would prefer not to conduct temporal statistic on per-county incidence because of limitations of the dataset. We lack personal identifiers (e.g., age and sex) which are potential confounders when comparing cumulative incidence. For example, because this disease primarily occurs in children, if the age distribution changed during the study period, then cumulative incidence may increase or decrease accordingly. To this point, we changed our language to focus less on temporal changes in incidence in an attempt to redirect the focus towards the geographic distribution of disease clusters.

We considered the emerging hotspot analysis tool in ArcGIS Pro thanks to your suggestion, but ultimately determined that it is not suitable for our dataset. Because our dataset is at the annual scale, we do not have a sufficient temporal resolution, as this method works best when there are many time steps (e.g., daily COVID-19 infections). At best, we could use this tool to identify “Persistent Hot Spots” and “Oscillating” or “Diminishing” hot spots, consistent with our findings in the original manuscript (i.e., persistent hot spots in Appalachia and oscillating hotspots in the upper Midwest). However, we prefer scanning statistics because local measures of spatial autocorrelation like the Getis-Ord Gi* used in ESRI’s Emerging Hot Spot Analysis have the problem of multiple testing, inflating the type I error rate. Still, this analysis seems to be an excellent tool for a dataset with a greater temporal resolution, and we will continue to explore its utility in future studies.

**Methods**

In the attached document, the reviewer suggested a change in the Ethics Statement. In response, we changed our wording in the statement to include “private health information”.

**Results**
*The study findings are logical and evidence-based. The study is distinct from the current scientific literature.*

Thank you.

**Conclusions**

*The conclusions are logical and evidence-based.*

Thank you.

**Editorial and Data Presentation Modifications?**

 (No Response)

**Summary and General Comments**

(No Response)

**Reviewer 3:**

**Summary and General Comments**

*I believe these findings would be valuable to the general body of knowledge regarding La Crosse virus disease. Most of the methods seem sound and the use of maps for data visualization is really effective. However, there are some methods and comparisons that I strongly disagree with and the writing is not clear and concise (see attached document for details).*

**Title**

*The title could be more concise. Consider removing “socioeconomically disadvantaged” from the title, I think it takes away from the main message. I also recommend adding the time frame to the title.*

As suggested, we revised the title, removed “socioeconomically disadvantaged”, and added the time frame.

**Abstract**

*Line 19: it should be noted that the authors are discussing reported LACV disease which may be different than the actual distribution of disease. Changes in awareness, diagnostic capability, and reporting practices could also contribute to the changes in reported disease geographic distribution.*

We agree with this statement. We added the word “reported” to the sentence in question and elaborated on reporting bias in the discussion section.

*Line 29: I disagree with the interpretation starting in this line. The analysis found association with higher incidence at the county level with increased proportions of White residents and lower median household income, not “high disease risk.” Please clarify the interpretation here.*

We changed “high disease risk” to “risk was higher…”

**Author Summary**

*Line 38: this statement should be amended to “pathogen that can cause neuroinvasive disease” as LACV infection can also be asymptomatic or cause febrile illness.*

We amended this sentence as suggested.

*Line 42: again, should be “reported LACV disease”*

We corrected this sentence.

*Line 47: “high-risk clusters of neuroinvasive LACV disease risk” doesn’t really make sense. I believe if you remove the second “risk” it would make sense.*

We corrected this sentence.

*Line 50: same feedback as line 29. The authors seem to be over-interpreting these results.*

We corrected this sentence.

*Line 52: please specify the time period you are referring to, as you’ve also stated that geographic distribution changed in the 1990s.*

We corrected these periods.

**Introduction**

*Line 61: I feel that it is important to also note that LACV infection can be asymptomatic (I believe the authors mention this in the discussion) and can also cause febrile illness. Also, the word “pediatric” is unnecessary and misleading here, I recommend removing it.*

We added a sentence explaining that LACV infections can be asymptomatic and cause febrile illness, but we kept the term “pediatric”. WNV causes more neuroinvasive disease in the US than LACV, but LACV causes more pediatric neuroinvasive disease. See Gaensbauer et al. (2014), who showed that from 2003 to 2012, WNV caused 97% of neuroinvasive arboviral disease, but WNV caused only 41% of pediatric cases. LACV caused 55% of pediatric cases.

*Line 63: again, I recommend specifying “reported” LACV disease cases.*

We added the word “reported”.

*Line 67: I’m not sure if this statement is factual, as Ohio has consistently had some of the highest reported case numbers for this time period.*

We agree that the sentence was misleading in the submitted manuscript. We added Ohio to the list of states in this sentence.

*Line 68: recommend specifying nationwide geographic clustering here, as Haddow et al. have another paper from 2011 with spatial analysis of LACV restricted to WV.*

We agree with this suggestion, but the revised manuscript does not include this sentence.

*Line 72: I know that Appalachia is depicted in the Figure 1 map, but it might be good to define where that region is earlier in the manuscript, especially given the international audience of PLOS Neglected Tropical Diseases.*

Thank you for pointing this out. We considered describing Appalachia in the introduction, but it did not fit well in the narrative. In the revised manuscript, we focused less on Appalachia in the introduction to avoid confusing readers before they see Fig 1.

*Line 79: I recommend deleting the clause from “As of this writing, …available in ArboNET” as it is misleading. You’ve just stated that LACV became notifiable in 2003, you are presenting data for 2003-2021, and we can’t really compare pre and post required reporting data periods.*

We revised this paragraph to clarify the time period of our study.

*Line 87: I would take out the individual time periods here since you will be presenting them in the methods section. Additionally, I don’t think you present your predictors analysis by those individual time periods.*

We removed the time periods from this sentence.

*Line 89: this occurs throughout the manuscript, but I believe this is the first instance. “Incidence risk” is a bit of a redundant term that isn’t used consistently in epidemiology. I believe the authors mean cumulative incidence which is a much more widely used term. I recommend changing “incidence risk” to “cumulative incidence” throughout. Also, the authors refer to their GLMM analysis as “predictive” throughout – can they clarify if this is really meant to be predictive modeling? Predictive feels like a strong conclusion to make from this analysis.*

We chose the term incidence risk because it is consistent with other terms in the manuscript (“risk ratio”, “risk cluster”) for which the calculations are fundamentally based on cumulative incidence. However, in response to this comment, we changed incidence risk to cumulative incidence throughout the manuscript and defined the other terms in the context of cumulative incidence.

Regarding the GLMM, we did not feel that the manuscript indicated that the GLMM was anything more than a standard approach to identifying “predictors”/risk factors/associated variables, but we changed the terminology to “associated” in response to these comments and clarified the limited applications of our model in the discussion section.

**Methods**

*Line 92: can the authors clarify why they restricted to these 31 Eastern US states?*

We added text describing the fact that nearly all LACV disease is reported from this study area.

*Line 97: can the authors clarify why only the 4 states with consistent high risk clusters were included in this analysis? What is the methodological reason behind excluding those other states?*

We excluded those states because they have few or no cases for most of the time period. Because the lack of reported disease is those states is probably due to factors not included in our analysis, we felt that including those states would create an issue of zero-inflation without providing useful associations with risk within states where the disease is consistently reported. We added text to the methods clarifying this point.

*Line 98: I recommend that the authors put in parentheses in this sentence where the definition of high-risk clusters can be found in the methods? This term has been used multiple times without a definition and it may be unclear for readers who are unfamiliar with spatial analytic methods.*

We better defined “cluster” in the “Geographic Cluster Analysis” section of the methods and referred to that section in “Methods: Study area and period”.

*Line 99: looking at the ArboNET case definitions this is a really oversimplified, recommend adding in more detail about the laboratory criteria specifically.*

We expanded on the laboratory criteria.

*Line 112: I don’t understand the sentence beginning in line 112 “To account for…time periods.” Can the authors re-write this sentence?*

We revised the sentence to clarify our meaning.

*Lines 116 – 121: this should be in the methods section titled “Predictor Investigation” as these are the variables included in those models. Additionally, table 3 also lists age as a predictor variable, please ensure that all variables from models are also in methods.*

We moved this to the “Predictor Investigation” section and added the age variable.

*Table 1: why was the 2009 ACS used for the 2003-2007 time period predictors?*

We used the 2009 ACS because other than the 2000 decennial census, the 2009 ACS was the earliest available survey that contained the predictor variables we aimed to use.

*Line 129: I question the validity of comparing mean county-level incidence over time or between states. Mean county incidence will be affected by how focal reported disease is in that state (i.e., every county that reports zero cases will dramatically skew the mean). For comparing over time and between states, I recommend calculating cumulative incidence for the entire state for each time period. County-level incidence is appropriate for maps such as Figure 2 and calculating spatial statistics. Additionally, in line 129 I believe the authors mean “among states across time periods.”*

We agree that mean county incidence is affected by how focal reported disease is in that state, but we think that state incidence is even more biased by the focal nature of this disease. In response to your comments, we replaced the mean county incidence with statewide cumulative incidence, but note that this change actually made most of the values even smaller (i.e., biased more towards zero). When including the entire state population in the calculation, the calculation ignores excessively high risks in the subset of counties where most disease is being reported. We think it is important to make this point, because reports of LACV disease that only consider statewide cumulative incidence effectively ignore the fact that a small portion of the state is bearing most of that burden. In other words, it is a repeated problem in the same area which could be predictable.

Based on the reviewer’s comments, we changed our approach to this. We reported the state cumulative incidence as suggested, but also added a table containing the cumulative incidence within each high-risk cluster. Instead of comparing mean county incidence in the whole state to mean incidence among counties that reported cases, we draw comparison to the statewide cumulative incidence and the cumulative incidence within the clusters. This comparison is valid because the clusters tend to include the majority of cases reported within each state, and therefore are a more accurate description of where the risk is present. We hope that the reviewer will agree with these revisions.

*Line 133: Can the authors add in a sentence or two with a more lay-person’s description of what these analyses results mean? Something like “to identify high-risk clusters – that is, groups of counties that have a much higher cumulative incidence of disease than the rest of the study area.” Many people will not be familiar with geospatial analytic methods and will be unable to understand your results without some more basic explanation.*

Thank you for this suggestion. We added a paragraph that describes this analysis in more detail and defines clusters as areas with significantly higher cumulative incidence than the overall study area.

*Line 140: please list what was used as the comparison group for calculation of relative risk.*

We added text that defines the comparison group (the cumulative incidence in the overall study area).

*Line 146: please list in results whether any variables were excluded because of this collinearity analysis.*

We added the variables which were excluded in the results. Thank you for noticing this error.

*Line 148: for the dependent variable please specify whether this was the incidence for the entire study period.*

We clarified that it is the number of cases grouped by time period.

**Results**

*Line 175: please give units for incidence (likely per 100,000 population). Additionally, it would be helpful to give the cumulative incidence for the entire study area for this time period so readers have some context for how high the incidence is in WV, NC, TN, OH, and WI. Lastly, I think it would be really helpful to include the cumulative incidence for each state mentioned here in parentheses so readers don’t have to reference the table.*

We the range of statewide CI for the four time periods along with CI in the total study area. We also added the CI in the total study area to Table 2 for easier comparison.

*Line 178: It doesn’t look like this information is included in Figure 1 as it says here. Please double check.*

We referenced Figure 1 because it informs readers of where the specific states we are referring to are located. In response to your comment, we removed the reference to figure 1.

*Line 180: Strongly recommend deleting the sentence “When including only the counties…included (Table 2).” This isn’t really a finding, it is just a mathematical fact that when you take 0’s out the mean will increase substantially. If the authors want to communicate how focal reported cases were in each state, I would recommend including the number and proportion of counties in each state that reported cases in the study period. I think that is a clearer way to communicate this concept.*

We removed this sentence because we changed our approach to comparing incidence for the whole state and for the focal area of risk (see comments above). However, while your reference to the mathematical fact is true, the fact that so many counties reported zero cases is significant and important for this disease system. If one removed all of the counties that reported no cases of COVID-19 in 2021, the mean county incidence would likely remain unchanged; because of the focal nature of LACV disease, removing counties with no cases has a large effect. We feel that it is important to highlight the fact that for LACV disease, statewide measures of risk/incidence can severely underestimate focal risk in the small portion of states like NC, where almost every case is reported in the southwestern region of the state.

*Table 2: Again, I advise against using mean county-level incidence and instead recommend calculating incidence for the entire state for each time period. The column names could also be cleaner.*

We revised this table as described above.

*Line 190: I believe the authors should remove “Counties with” here as the RRs are associated with the cluster, and not the counties.*

We revised our wording to refer to the clusters instead of counties.

*Line 196-201: the way these results are presented is confusing. The authors should consider reporting how many clusters were in each number of time periods – the use of “at least X periods” for some of them is confusing.*

We attempted to clarify the results as originally written. We do not feel that reporting instead the number of clusters in each period does not convey that several counties were repeatedly in high-risk clusters during the entire study period.

*Line 204: I strongly recommend including the estimates for measure of effect in this paragraph, along with an interpretation (e.g., a $1000 decrease in median income in a county is associated with an X% increase in neuroinvasive LACV cases in the county). From the manuscript as currently written it is unclear what the magnitude of association/effect is, and the IRRs, while significant, are close to one.*

We added risk ratios and their interpretation (now simply called risk ratio) in the text of the results. We agree that the added interpretation will clarify the magnitude of the effect. Here is the added sentence for your reference: “While holding the other predictor constant, a one-percent increase in the percentage of the population that is white was associated with an 8% increase in CI of LACV-ND (RR = 1.08), and a $1,000 increase in median household income was associated with a 3% decrease in the CI of LACV-ND (RR = 0.97).” Although the magnitude is close to one, the scales of the variables are also small. We feel that the measures of association are notable.

**Discussion**

*Line 223: this comparison should not be made. Firstly because one is incidence at the state level and one is mean county incidence in the state. Secondly, the mean county incidence presented here only includes counties that reported a case. This is not a valid comparison.*

We think it is important for readers to recognize that the burden of LACV disease is underrecognized when reported nationally or at the state level. The majority of reported LACV disease occurs in a subset of counties, many of which have small populations. State-level risks ignore the restricted distribution of disease, and completely negate the small populations in counties where most of the cases are reported. To this point, the revised manuscript contains our own calculations of state-level incidence risk for each time period to compare with the cumulative incidence within the clusters. Although the denominator (population) is different, we think the comparison is valid, especially because the clusters contain most of the cases within the states.

*Line 229: can the authors discuss how the high-risk clusters differed over time? In the results it is mentioned that clusters varied in size, shape, and that the clusters in Iowa/Wisconsin was only present in some of the analyzed time periods. Are there any possible explanations for that?*

We added a paragraph to the discussion section that addresses changes in clustering and cumulative incidence during the study period. There is no clear answer, but we suggest that increased human immunity, LACV resistance in mosquito populations, or confounding from the aging human population are possible contributors.

*Line 239: I think it is very important for the authors to also discuss that geographic distribution of LACV disease is highly dependent on distribution of vectors and amplifying hosts. These are complex factors that aren’t mentioned in this manuscript at all. Also, the authors mention here that distribution is highly predictable, but earlier mentioned the expansion into the Southeast in the 1990s. This is a contradictory statement – potentially the authors mean that geographic distribution has been stable for this time period? I think that’s as far as we can conclude based on this data.*

We agree that we were not clear about the vectors and hosts within the text and added it to the manuscript. The vectors and amplifying hosts are widely distributed throughout the eastern United States. We added text to the discussion explaining that the distribution of vectors and amplifying hosts is insufficient to explain the restricted distribution of LACV disease in this study area.

Regarding the predictable distribution of the disease – this was not the point we intended to make, although we would argue that nearly 20 years of a stable distribution does imply predictability. We revised this statement to clarify that our study builds on previous evidence that locations where LACV transmission occurred in the past are likely to be areas of high risk in the future (i.e., spatially persistent risk). We want to support the idea that targeted prevention efforts may be effective for preventing future disease.

*Line 244: why was the GLMM analysis restricted to those 4 states? Is there some thought that there might be different factors associated with LACV risk in other states? Also, in this section the authors make too strong of conclusions based on their findings – I think it’s fair to say that there was an association between the two variables and case numbers at the county level, but maybe not that risk can be predicted by these two variables.*

We restricted the analysis to those four states because the other states reported very few or zero cases. This point was added to the methods section. We also changed our wording to “associated with” but note that there is no defined effect threshold for a predictor variable to be considered predictive of risk.

*Line 252: the word “treatment” is included in this sentence twice*

Thank you, we removed the duplicated word.

*Line 270-282: the limitations paragraph is unclearly written. For example, in the second sentence there are two facts listed but it is unclear how these facts could affect the validity of these findings. The third sentence I believe is alluding to bias from awareness, but it is unclear. Also, there are some limitations that are not addressed: only variables previously associated to be associated with increased LACV risk were included in the GLMM model, so some risk factors might be missed, the GLMM model only included 4 states, so those findings are not generalizable beyond those states, and the temporal analysis did not use statistical methods, so temporal analysis is based on observation and descriptive statistics.*

We revised this paragraph to help readers connect the listed limitations to the results of our study. We also added a sentence stating that there may be other risk factors which were not considered here. We mention the lack of generalizability earlier in the discussion section.

*Line 276: please clarify – ArboNET does collect demographic data for cases, but it was just not available in the dataset used for this analysis.*

The submitted manuscript did not imply that ArboNET does not collect demographic data. As stated, they do not offer those data at the county level.

**Conclusions:**

*Line 290: please specify that it is persistent in Appalachia since the 1990s. Also recommend adding “economically” in front of disadvantaged communities since your findings only found White race and lower median household income to be associated with county level risk.*

We specified the time period of the study in the conclusion.
